# 

**Published:** 1908-07-11

**Authors:** 


### BOOKS RECEIVED.

The Scientific Press, Ltd.

"The Heart and Sudden Death." By Theodore Fisher, M.D.

"The Pilot" Press.

"Cancer: Operation not the Cure but a Cause." By J. Shaw, M.D.

J. and A. Churchill.

"The Practice of Medicine." By Frederick Taylor, M.D. 8th Edition.

Cassell and Co., Ltd.

"Clinical Methods." 4th edition. By R. Hutchison, M.D., and H. Rainy, M.D.

"Hygiene and Public Health." By B. A. Whitelegge, C.B., M.D., and G. Newman, M.D., D.P.H.

### THE GRADUATES' MEDICAL SCHOOLS.

DIARY FOR WEEK JULY 13 TO 18.

**THE HOSPITAL FOR SICK CHILDREN, Great Ormond Street, W.C.**

**At 4 p.m.**
**July 16,** Dr. Hutchison, **Congenital Pyloric Stenosis.**

**THE POST GRADUATE COLLEGE, West London Hospital, Hammersmith, W.**

**At 12 noon.**
**July 13,** Dr. Low, **Pathological Demonstration.**
**At 12.15 p.m.**
**July 15** and **17,** Dr. Pritchard, **Practical Medicine.**
**At 5 p.m.**
**July 13,** Dr. Saunders, **Clinical.**
**July 14,** Mr. Pardoe, **The Differential Diagnosis of Scrotal Swellings.**
**July 15,** Dr. Beddard, **Medicine—VI.**
**July 16,** Mr. Dunn, **Cases of Eye Disease.**
**July 17,** Dr. Saunders, **Clinical.**

**MEDICAL GRADUATES' COLLEGE AND POLYCLINIC, Chenies Street, W.C.**

**At 5.15 p.m.**
**July 13,** Mr. C. R. Keetley, **Affections of the Toes, Feet, and Ankles.**
**July 14,** Dr. Robert Jones, **Insanity, Wit, and Humour.**
**July 15,** Mr. C. H. Leaf, **The Principles underlying Surgical Treatment in so-called Inoperable Cancer.**
**July 16,** Dr. H. D. Rolleston, **The Association of Jaundice and Splenic Enlargement.**

**LONDON SCHOOL OF CLINICAL MEDICINE, Seamen's Hospital, Greenwich, S.E.**

**At 4 p.m.**
**July 13,** Mr. Lawrence, **Hearing.**
**At 2.15 p.m.**
**July 14,** Dr. Hewlett.

THE HOSPITAL *July* 11, 1908.

*Name* ..............................................................

*Address* ..............................................................

..............................................................

This Coupon must accompany manuscript or contributions intended for The Hospital.

